# Near-surface Heating of Young Rift Sediment Causes Mass Production and Discharge of Reactive Dissolved Organic Matter

**DOI:** 10.1038/srep44864

**Published:** 2017-03-22

**Authors:** Yu-Shih Lin, Boris P. Koch, Tomas Feseker, Kai Ziervogel, Tobias Goldhammer, Frauke Schmidt, Matthias Witt, Matthias Y. Kellermann, Matthias Zabel, Andreas Teske, Kai-Uwe Hinrichs

**Affiliations:** 1 MARUM Center for Marine Environmental Sciences, University of Bremen, Leobener Strasse, D-28359 Bremen, Germany; 2 Department of Oceanography, National Sun Yat-Sen University, 80424 Kaohsiung, Taiwan; 3 Alfred-Wegener-Institut Helmholtz Zentrum für Polar und Meeresforschung, Am Handelshafen 12, D-27570 Bremerhaven, Germany; 4 Department of Marine Sciences, University of North Carolina at Chapel Hill, Chapel Hill, 27599 NC, USA; 5 Leibniz Institute of Freshwater Ecology and Inland Fisheries, 12587 Berlin, Germany; 6 Bruker Daltonics GmbH, Bremen, Germany

## Abstract

Ocean margin sediments have been considered as important sources of dissolved organic carbon (DOC) to the deep ocean, yet the contribution from advective settings has just started to be acknowledged. Here we present evidence showing that near-surface heating of sediment in the Guaymas Basin, a young extensional depression, causes mass production and discharge of reactive dissolved organic matter (DOM). In the sediment heated up to ~100 °C, we found unexpectedly low DOC concentrations in the pore waters, reflecting the combined effect of thermal desorption and advective fluid flow. Heating experiments suggested DOC production to be a rapid, abiotic process with the DOC concentration increasing exponentially with temperature. The high proportions of total hydrolyzable amino acids and presence of chemical species affiliated with activated hydrocarbons, carbohydrates and peptides indicate high reactivity of the DOM. Model simulation suggests that at the local scale, near-surface heating of sediment creates short and massive DOC discharge events that elevate the bottom-water DOC concentration. Because of the heterogeneous distribution of high heat flow areas, the expulsion of reactive DOM is spotty at any given time. We conclude that hydrothermal heating of young rift sediments alter deep-ocean budgets of bioavailable DOM, creating organic-rich habitats for benthic life.

The vast pool of marine DOC represents an important growth substrate for heterotrophic microorganisms^1^. In the deep ocean, DOC is considered to be refractory^2^, yet recent carbon budgets in the bathypelagic zone suggested that ‘dark ocean respiration’, driven mainly by prokaryotic activities^3^ below the euphotic zone, frequently exceeds the total influx of organic carbon (including particulate organic carbon and DOC) from the upper ocean^4^. One of the explanations for this imbalance is the existence of unaccounted sources introducing biodegradable organic matter into the deep ocean^4^. Although it has been argued that ocean margin sediments are quantitatively important sources of DOC to the deep ocean^5^,^6^, the contribution from advective settings, such as cold seeps^7^, has just started to be acknowledged.

Thermally altered sediments have not been accounted for as benthic DOC sources. Laboratory experiments and field studies have demonstrated marked accumulation of bulk DOC and acetate, a common substrate for prokaryotic life, in diagenetically heated marine sediment^8^,^9^,^10^. Although the geothermal gradients of 30–80 °C km^–1^ (ref. 11) in most sedimentary basins require great subseafloor depths to trigger this process, near-seafloor heating of sediment also takes place in spreading centers. To examine the flux of DOC and biodegradability of DOM from such environments, we measured the DOC concentration in the bottom water and sediment from the Guaymas Basin (Table 1 and Fig. S1 of Supplementary Information), an extensional depression representing the transition from continental rifting to seafloor spreading^12^. In addition, batch experiments simulating low-temperature heating were carried out to study the formation and composition of heat-mobilized DOM. These experimental data were finally used to estimate the DOC efflux from the young rift sediment at different spatiotemporal scales. Our multiple lines of evidence indicate that near-surface heating of young rift sediment creates mass production and release of reactive DOM, a process that has not been reported in other seafloor environments.

## Results and Discussion

### Sediment geochemistry and water-extractable DOC (DOC_WE_)

**Figure 1:**
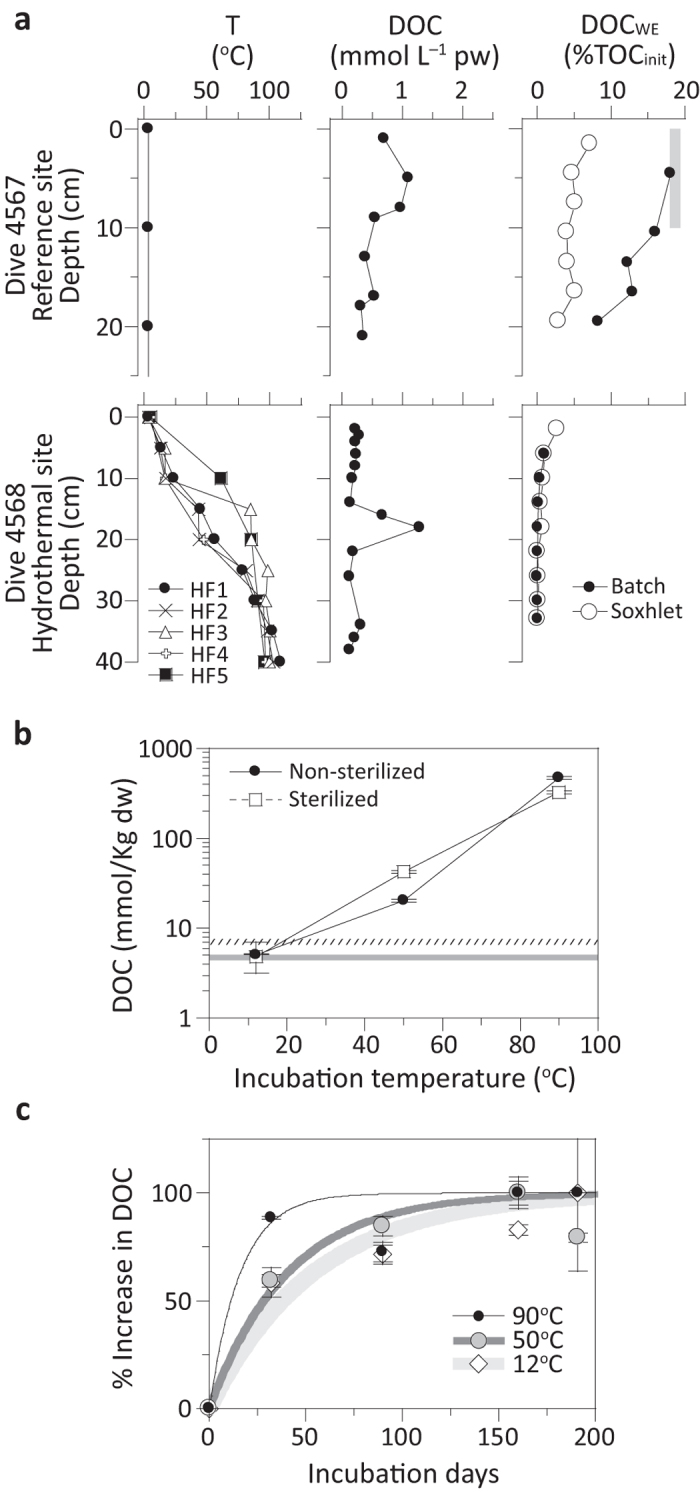
(**a**) Temperature from the heat flow (HF) measurements, DOC in the pore water (pw), and DOC_WE_ of the reference and hydrothermal sites from *Alvin* Dives 4567 (core 27) and 4568 (core 1). DOC_WE_, determined by two extraction methods and reported as the fraction relative to the initial total organic carbon content (TOC_init_), represents the organic carbon pool that could be readily mobilized from the solid phase. TOC_init_ was set to be 3% dw based on the measured value of the top 5 cm sediment from the reference site (Fig. S3 of Supplementary Information). The final yield of DOC in the non-sterilized samples after prolonged incubation at 90 °C, as presented in (**b**), is also marked (gray shade) for comparison. (**b**) DOC concentrations in the slurries of the heating experiments after 191 days of incubation. The results are presented as mean and range of duplicate incubations. Grey-shaded bar, time-zero value of non-sterilized sediment slurry; shaded bar with slashes, time-zero value of the sterile control. (**c**) Accumulation of DOC over time in the experiments of non-sterilized sediment slurries at 12, 50 and 90 °C. The DOC concentrations were normalized to the maximum DOC concentration detected at each temperature during the incubation. The data, presented as mean and range of duplicate incubations, were broadly fitted with first-order kinetics.

In the hydrothermally altered sediment, the temperature increased from 3 °C to over 100 °C within less than 40 cm (Fig. 1a). Using Fourier’s law and thermal conductivity of 0.9 W m^−1^ K^−1^ for the bulk sediment, we obtained an average heat flow of 207 W m^−2^. Neither did the hydrothermal sites show elevated levels of DOC relative to the reference site (Fig. 1a; see Fig. S2 of Supplementary Information for the profiles from other hydrothermal sites; the reference site is published in McKay *et al*.^13^), nor did DOC concentration profiles increase exponentially with depth as typically observed in anoxic, organic-rich sediment in ocean margins^6^. The seawater-level content of Mg^2+^ in pore waters of the hydrothermally impacted sediment and the presence of ample sulfate (22‒26 mmol L^−1^ pore water) despite the high sedimentary sulfur content (3‒6.5% of dry weight (% dw); Fig. S3 of Supplementary Information) imply advective pore fluid flow, possibly driven by hydrothermal fluid circulation between shallow sediment and seawater^14^. Similar to previously published push cores from the Guaymas Basin hydrothermal field^15^, sediment from the heated site was heavily impregnated with petroleum and exhibited total organic carbon contents as high as 5‒8% dw (Fig. S3 of Supplementary Information). When the bituminous material was removed via solvent extraction, the heated site, especially in sediment deeper than 5 cm, possessed only half of the kerogen carbon content observed at the reference site. This data set indicates that thermally induced loss of organic matter from the solid phase and the subsequent migration of heat-mobilized products have occurred. With rising temperature, kerogen rearranges its structure and releases mobile organic compounds^16^. A fraction of these compounds is oily, condenses on cold particle surfaces when migrating upward, and forms the oil-impregnated sediment that is characteristic for Guaymas Basin^17^,^18^,^19^.

In order to test whether such a “mobilization and expulsion” mechanism also applies to water-soluble compounds, we measured water-extractable DOC (DOC_WE_), the organic carbon pool that could be readily mobilized from the solid phase by short-term aqueous extraction. We employed two methods, batch extraction with artificial seawater and Soxhlet extraction with Milli-Q water, to constrain the range of DOC_WE_ contents. Both methods generated the same pattern of much lower DOC_WE_ contents by one to two orders of magnitude in the heated site compared to the reference site (Fig. 1a), consistent with the prediction of thermal mobilization and expulsion of DOC from the sediment under *in situ* conditions. In addition, we did not see indications of DOC condensation in the sediment of the heated site, suggesting that expulsion into the water column or degradation represent the major fates of mobilized DOC. Taken together, the geochemical data suggest that DOC distribution was not controlled by diffusion but by hydrothermal mobilization and circulation. The heat-driven fluid flows through the sediment flushes away solutes released from the organic-rich sediments, gradually depletes the mobilizable fraction of organic matter on the particle surface over time, and finally decouples the extant pore-water DOC distribution from the temperature regime.

### Formation and composition of heat-mobilized DOM

Since extant pore-water profiles of DOC are impacted and altered by advective fluid flow, they cannot be used to assess the efflux and degradability of sedimentary DOC upon heating. We instead studied the formation and composition of heat-mobilized DOC by means of long-term (191 days) heating experiments using the reference site sediment. Both non-sterilized and sterilized sediments showed an exponential increase in DOC with temperature (Fig. 1b), confirming the predominantly abiotic nature of heat-induced DOC release. The marked difference in DOC between the non-sterilized and sterilized sediment incubated at 50 °C reflected the activity of heterotrophs, which also resulted in a substantial decrease of sulfate and accumulation of dissolved inorganic carbon (see Fig. S4 of Supplementary Information). Accumulation of DOC over time in the heating experiments can be broadly fitted with first-order kinetics (Fig. 1c). In the heated sediment (50 and 90 °C), the maximum DOC concentrations were reached within only dozens of days, suggesting thermal mobilization of DOC from sediment to be an exceptionally rapid process compared to diagenetic alteration and remineralization.

**Figure 2:**
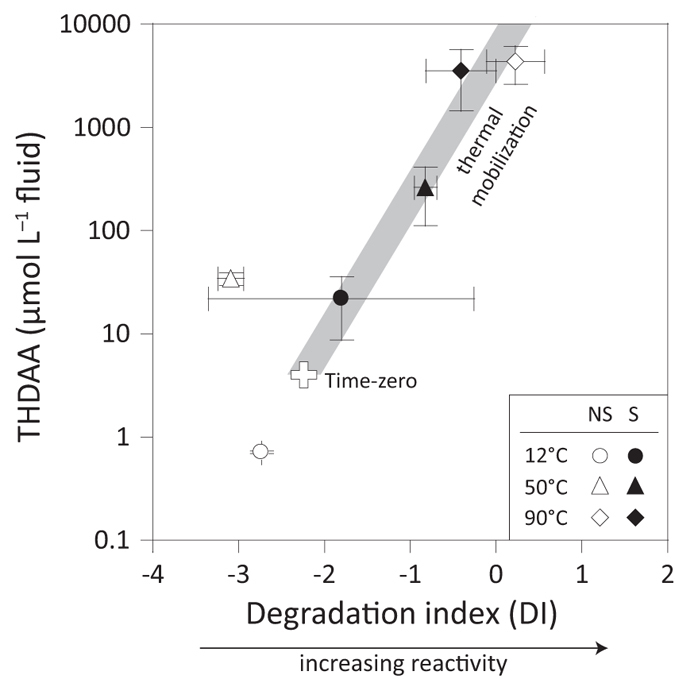
Concentrations of THDAA in the slurries of the heating experiments after 191 days of incubation, and the corresponding DI values(cf. Dauwe *et al.*^20^). The gray line delineates the abundance and compositional change of THDAA with temperature. The two non-sterilized samples falling out of the gray line manifest the effects of biodegradation at lower temperatures on the concentration and compound distribution of the THDAA. The results are presented as mean and range of duplicate incubations. NS, non-sterilized sediment slurries; S, sterilized slurries.

The degradability of heat-mobilized DOM was assessed with total hydrolyzable dissolved amino acids (THDAA), which also increased exponentially from 0.7 to ~4300 μmol L^−1^ fluid with elevated temperature (Table 2 and Fig. 2). The observed increase of THDAA-based N to total dissolved nitrogen during heating of sterilized samples, from 5.9% at 12 °C to 20% at 90 °C (Table 2), provides a first indication of enhanced reactivity. The degradation index (DI; ref. 20) was calculated using the concentration data of 14 amino acids (see Table 2). More positive values indicate greater reactivity of organic matter. For the sterilized sediment, which showed a fairly linear trend on a double logarithmic scale, the elevated DI at 50 and 90 °C was mainly ascribed to the selective enrichment of leucine and isoleucine, which together contributed to a positive shift of 0.8‒1.2 along the DI scale. The heat-mobilized THDAAs were biodegradable, as manifested by a decrease of 87% in THDAA concentration in the non-sterilized sediment at 50 °C compared to the sterilized control. This biodegraded, residual DOM, enriched in aspartic acid and depleted in leucine, isoleucine and methionine, exhibited the lowest DI and hence the highest degree of recalcitrance.

**Figure 3:**
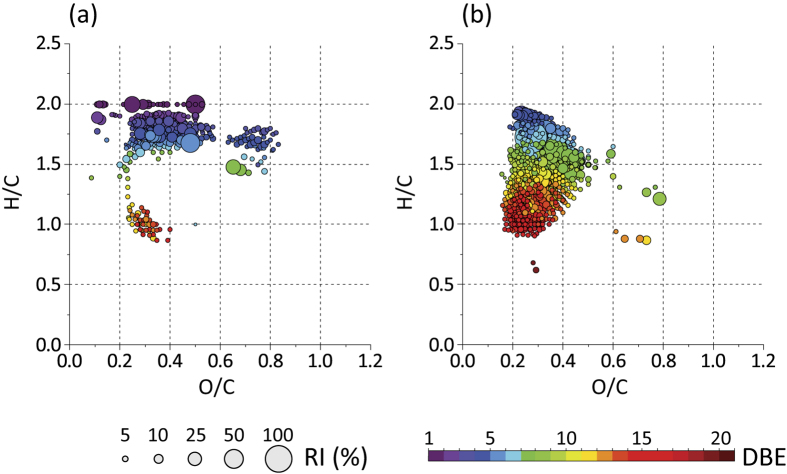
Negative electrospray ionization FT-ICR-MS data of heat-activated DOM in the 90 °C non-sterilized incubations, presented as van Krevelen diagrams. Heat-activated DOM is defined as those formulas present only in the heated sample but absent in the time-zero and 12 °C samples. (**a**) CHO species. (**b**) CHON species with three to four nitrogen atoms. The bubble color indicates double bond equivalent (DBE), and the bubble size denotes relative intensity (RI).

Non-targeted molecular characterization of heat-mobilized DOM using Fourier transform-ion cyclotron resonance-mass spectrometry (FT-ICR-MS) further revealed the presence of molecular formulas that can be affiliated with reactive compound classes (Fig. 3; see also Table S1 and Fig. S5 of Supplementary Information). Compared to low-temperature samples, the 90 °C sample exhibits selective enrichment of CHO and CHN_3–4_O species with high H/C and low O/C ratios (see Table S1 of Supplementary Information). Two groups of CHO species became detectable only after heating of the sediment (Fig. 3a). The formulas of the first group (O/C > 0.6, H/C > 1.3) resembled the elemental composition of carbohydrates^21^, whereas the other group was less oxidized but more aliphatic (O/C < 0.6, H/C > 1.7, double bond equivalent ≤4)^22^. About 30% of the heat-activated aliphatic compounds shared the same molecular composition as the metabolites recently found in oil-entrapped water droplets^23^, suggesting their possible identity as activated hydrocarbons^24^. The most striking feature of heat-mobilized DOM is the elevated proportion of CHN_3‒4_O formulas, 34% of which matching the composition of tri- and tetrapeptides (Fig. 3b and Fig. S6 of Supplementary Information). These lines of evidence agree well with the THDAA data (Fig. 2) and reinforce the argument of reactive heat-mobilized DOM.

The compositional data also suggest that multiple mechanisms besides desorption, which is known to be highly temperature-dependent^25^, are shaping the molecular composition of DOM. For example, the detection of molecules affiliated with activated hydrocarbons suggests that hydrophobic compounds become more accessible to microorganisms at elevated temperature, possibly via their enhanced solubility in hot water^26^. The presence of the identified oligosaccharide- and oligopeptide-like components would require hydrolysis of biomacromolecules to smaller fragments in the heated sediment^27^. An in-depth examination of the molecular composition and mobilization mechanism of DOM is beyond the scope of this study and will be presented elsewhere.

### Assessing the DOC efflux

To estimate the contribution to deep-sea DOC from near-surface heating of young rift sediment, the heat-induced sedimentary DOC efflux needs to be constrained. We first employed a simple reaction-transport model to obtain insights into the magnitude and evolution of transient DOC efflux upon a heating event. Three parameters that have been experimentally determined in the present work were incorporated into the model. First, the temperature dependence of maximum DOC yield can be derived from the results of the non-sterilized incubations (Fig. 1b):


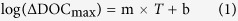


where ΔDOC_max_ (μmol g^−1^ dry sediment) is the difference between maximum and initial DOC concentrations, *T* is the experimental temperature in °C, and m and b are coefficients determined by linear regression.

The DOC evolution records at three incubation temperatures (Fig. 1c) enabled us to mathematically describe the second parameter, the kinetics of DOC production:


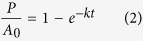


where *P* is the concentration of product as a function of time, *A*_0_ the initial concentration of reactant, *k* the rate constant, and *t* the reaction time. In our case, we set *A*_0_ as ΔDOC_max_ and *P* as ΔDOC_*t*_, which is the concentration difference in DOC (in μmol g^−1^ dry sediment) between a specific time point and experimental start. *k* can be further fitted by the Arrhenius equation:


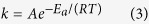


where *A* is a prefactor, *E*_*a*_ is the activation energy, and *R* is the universal gas constant.

The advection of warm fluids creates temperature anomalies (Fig. 1a), which in turn provide information on the third parameter, the advection rate of pore water. Assuming steady flow with a constant source temperature, the resulting temperature profile is described by the following analytical solution to the heat transfer equation (modified from Henry *et al*.^28^):


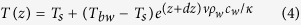


where *z* is the subseafloor depth (positive downward), *dz* the depth offset to compensate for inaccurate estimates of penetration depth, *T*_*s*_ the source temperature, *T*_*bw*_ the bottom water temperature, *v* the Darcy velocity (upward is negative because *z* is positive downward), *ρ*_*w*_ the density of pore water, *c*_*w*_ the specific heat capacity of pore water, and *κ* the thermal conductivity of the bulk sediment.

**Figure 4:**
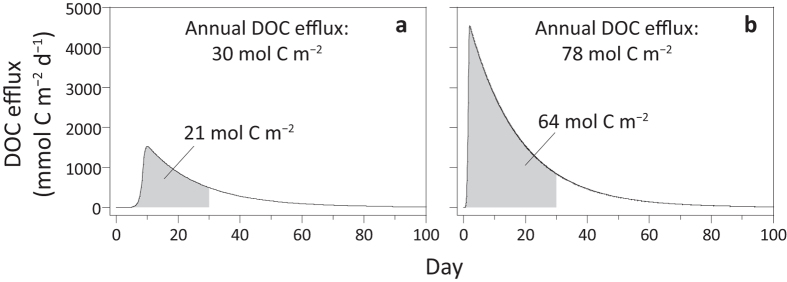
Simulated efflux of DOC from sediment upon a heating event with the temperature regime represented by (**a**) HF1 and (**b**) HF5 of Dive 4568. The shaded areas represent the cumulated DOC efflux in the first 30 days after heating.

**Figure 5:**
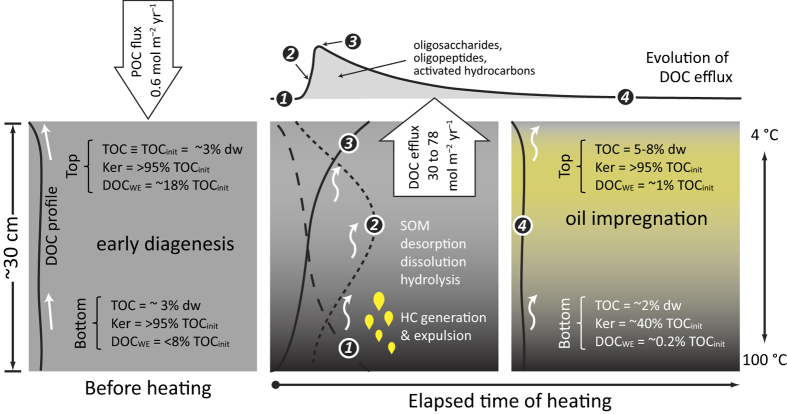
A schematic diagram summarizing the effects of near-surface heating on mobilization of sedimentary organic matter (SOM) and the consequential massive discharge of reactive DOM and impregnation with oil-like substances. The straight and curved white arrows denote diffusion- and advection-dominant solute transport, respectively. Abbreviations: HC, hydrocarbon; Ker, kerogen; TOC, total organic carbon.

The values of parameters, coefficients and constants are listed in Table S2 of Supplementary Information. Equations (1) to (4) and the diffusion coefficient of DOC^29^ were used in the reaction-transport model Explicite^30^ to simulate the temporal evolution of DOC efflux. In the case of the hydrothermal site visited in Dive 4568, the modeled DOC efflux from the upper 33 cm of heated sediment was 30 to 78 mol C m^–2^ yr^–1^ (Fig. 4). The production of DOC was temporally heterogeneous; more than 70% of the heat-mobilized DOC would be flushed out to the bottom water in the first 30 days after heating, leading to instantaneous efflux as high as 4.5 mol C m^–2^ d^–1^. The bottom-water DOC concentrations reflected the impact of the short and massive DOC discharge events: despite the presence of strong bottom currents with velocities up to 12 cm s^–1^ (ref. 31), the seawater sampled 2 m above the seafloor (Table 1) had DOC concentrations 3 to 50 times higher than the typical concentration (~40 μmol L^−1^; ref. 2) in the bathypelagic zone. The effects of near-surface heating on mobilization of sedimentary organic matter and the consequential massive discharge of reactive DOM and concurrent impregnation of the sediment with oil-like components are schematically summarized in Fig. 5. The DOC efflux from a heated, 30-cm thick sediment column exceeds the particulate organic carbon burial flux from the overlying water column^32^ by two orders of magnitude. This imbalance occurs because the heat-mobilizable fraction of sedimentary organic matter that has accumulated over hundreds of years is released during comparatively short periods of heating.

For longer time scales, in the presence of a temporally stable heat source, the availability of heat-mobilizable organic matter is the main controlling factor of DOC efflux. To assess the long-term average DOC efflux for the basin, we adopted an approach following Lizarralde *et al*.^12^, who based their estimation of carbon release, irrespective of the form of carbon, on the sedimentation rates and efficiency of magmatism-induced alteration processes. Our long-term heating experiment showed that at 90 °C, the efficiency of DOC production was equivalent to up to 0.56% dw (or ~19% of the initial total organic carbon content of 3% dw; Figs 1a and S3), which translates to 28% of the maximum total carbon loss induced by magmatism^12^. After correcting for carbon loss due to background remineralization represented by the decline of DOC_WE_ contents with depth at the reference site (~6% of the initial total organic carbon content based on the average of the two DOC_WE_ data sets; Fig. 1a), we obtained a maximum DOC flux of 27 Gg C yr^–1^ based on a sedimentation rate of 0.18 cm yr^–1^ (ref. 33) and an area of ~4950 km^2^ of active sill intrusion^12^. This maximum flux needs to be revised based on the spatial heterogeneity of near-surface heating^13^, temporal fluctuation in fluid temperature^34^, and consumption of DOM by benthic organisms. Assuming that <1% of the sediment undergoes near-surface heating at the current stage, we estimated the DOC efflux averaged over the whole area of magmatism to be <5 mmol C m^−2^ yr^−1^, equivalent to <0.8% of the particulate organic carbon burial flux in the overlying water column^32^. For the majority of the sediment that has heat flow barely exceeding 10 W m^−2^ (ref. 35), the mobilized DOC at deeper depths will accumulate in the pore fluid^36^ due to the sluggish pore fluid flow^37^ and long transport distance to the seafloor. Therefore, the massive DOC discharge during spotty near-surface heating contributes little in counteracting the natural carbon sequestration in this highly productive coastal ocean, an implication highlighted by Lizarralde *et al*.^12^ for the shallow magmatism in young sedimented rift. However, if the young rift system of the Guaymas Basin continues to spread in a manner similar to nascent mid-ocean ridges, the extensional force would allow magma intrusion to occur at increasingly shallower depths^38^ in a widening basin, elevating the proportion of DOM released to seawater. Such a massive release of bioavailable DOM could stimulate respiration in the deep water at a basin-wide scale, thereby altering the bottom water oxygen content^34^,^39^, carbon chemistry, and even pH. Most importantly, this process creates unprecedented DOM-rich oases at the seafloor, making the young rift sediment a natural incubator of rich benthic life^34^,^40^.

## Material and Methods

### Sampling

Bottom-water samples in the Southern Trough of the Guaymas Basin were retrieved from ~2 m above the seafloor by 5-L Niskin bottles mounted on the Human Occupied Vehicle (HOV) *Alvin* during the expedition AT15–56 (November-December, 2009) of the *RV Atlantis* (Table 1 and Fig. S1 of Supplementary Information; see McKay *et al*.^13^ for detailed description). Push core sampling and heat probe measurements were also carried out by the HOV *Alvin*. Seawater samples were filtered through pre-combusted (450 °C, 8 hrs) glass fiber filters (0.7 μm) for DOC analysis. Pore-water samples for geochemical analyses were extracted by the Rhizon suction samplers^41^ (pore size 0.2 μm). Sediment samples were cut into 3- to 4-cm thick slices for solid phase analysis and aqueous extraction. Except for the supplementary pore-water DOC profiles (Fig. S2 of Supplementary Information), all of the analyses and experiments were based on materials from microbial mat-free sediment from Dives 4567 (reference site) and 4568 (hydrothermally impacted site). The distance between these two sites was about 200 m.

### DOC_WE_ analysis

The downcore DOC_WE_ distribution was determined via both batch extraction with artificial seawater and Soxhlet extraction with Milli-Q water. For batch extraction, about 10 to 30 g of frozen, wet sediment from each depth were mixed with artificial seawater at a ratio of 1:2.4 (w/w) to make bulk slurry. The slurries were purged with argon, dispensed into 20 mL glass ampoules, flame sealed, and incubated in the dark at 90 °C for up to 30 days. To harvest the aqueous phase for analysis, all slurry samples were allowed to cool down to room temperature before phase separation. Slurries in the ampoules were centrifuged and the supernatant filtered with Rotilabo polytetrafluoroethylene syringe filters (pore size 0.45 μm; Carl Roth GmbH, Karlsruhe, Germany). The fluid samples were stored at −20 °C until DOC analysis.

The Soxhlet extraction was carried out broadly following the procedure described in Schmidt *et al*.^27^ The Soxhlet extractors were cleaned by alkaline detergent and thoroughly rinsed with Milli-Q water prior to use. To reduce the blank DOC level, the assembled extractors containing empty Whatman glass microfiber thimbles (30 × 100 mm; precombusted at 450 °C for 8 hrs) were flushed with heated Milli-Q water for 24 hrs. After the cleaning step, 10 to 20 g of frozen, wet sediment were weighed into the thimbles and extracted with 200 mL of fresh Milli-Q in the dark for 48 hrs. The extracts were filtered through pre-combusted Whatman GF/F filters, weighed, and stored at −20 °C until DOC analysis. For the reference site sediment, Soxhlet extraction yielded lower DOC_WE_ than batch extraction by a factor of 2.5 to 3.9 (Fig. 1a), possibly due to the shorter extraction time, the lower reaction temperature (ca. 80 °C; ref. 27), the lack of ionic strength in the solvent, or the combination of these factors.

The measured DOC_WE_ values were normalized to the initial total organic carbon content (TOC_init_) of 3% dw, which was the measured value of the top 5 cm sediment from the reference site (Fig. S3 of Supplementary Information). This TOC_init_ value is in the range (2.3‒3.5% dw) reported for the surface sediments in the central Gulf of California^42^. Given the proximity of the reference and heated sites, we used the same TOC_init_ for samples from both sites.

### Heating experiments

Near-surface sediment (0–10 cm) and overlying water in the core taken from the reference site were pooled to make slurries for the heating experiment. These materials differed from those for DOC_WE_ analysis in that they were kept cold (4 °C) instead of frozen after the cruise. The sediment slurries were dispensed into customized 40 mL glass ampoules in an anaerobic chamber, flushed with argon, flame sealed, and incubated in the dark at 12, 50, and 90 °C for up to 191 days. Sterilized controls were prepared by adding zinc chloride into the slurries to a final concentration of 5% (w/v). To monitor the progress of reactions, a suite of small-volume (4 mL) slurries in small glass vials were incubated at each of the temperatures and were sacrificed sequentially for interim geochemical analyses. Because of material limitation, we had only duplicates for the large-volume incubations. To harvest the aqueous phase for analysis, all slurry samples were allowed to cool down to room temperature before phase separation. Slurries in the glass vials were centrifuged and the supernatant was filtered with syringe filters as described above. Slurries in the ampoules were extracted in an anoxic chamber with Rhizon suction samplers. The fluid samples for biogeochemical analysis were stored at −20 °C. The samples for non-targeted DOM analysis were immediately extracted (see below).

### Biogeochemical analysis

DOC and total dissolved nitrogen were measured with high-temperature catalytic oxidation-infrared detection. Solid-phase carbon contents were determined by an elemental analyzer. Sediment samples were first decalcified for measurements of total organic carbon content, and then treated by a mixture of dichloromethane and methanol (volume ratio = 9:1 v/v) for determination of kerogen carbon in the non-extractable residue. Sulfate concentration was determined by ion chromatography. DIC was determined as carbon dioxide liberated from acidified samples by infrared spectroscopy. Dissolved Mg^2+^ was determined by inductively-coupled plasma-optical emission spectroscopy. Total sulfur contents of the sediment samples were determined by an elemental analyzer. Fluid samples for THDAA analysis were acid hydrolyzed, pre-column derivatized with *o*-phthaldialdehyde and *N*-isobutyryl-L/D-cysteine, and analyzed with high-performance liquid chromatography-fluorescence detection^43^.

### FT-ICR-MS analysis

DOM for non-targeted mass spectrometric analysis was extracted using solid-phase extraction cartridges following the procedure described in Dittmar *et al*.^44^ Samples were ionized by an Apollo II electrospray source (Bruker Daltonik GmbH, Bremen, Germany) in negative ion mode and measured on a SolariX (FT-ICR-MS; Bruker Daltonik GmbH) equipped with a 12 T refrigerated actively shielded superconducting magnet (Bruker Biospin, Wissembourg, France). Data evaluation started with internal calibration of the mass spectra with a suite of compounds repeatedly identified in marine DOM samples^22^,^45^. The root mean square error of the internal calibration was below 0.100 ppm. Data evaluation was performed in the range m/z 200 to 600. For peaks with a signal to noise ratio higher than 3, molecular formulas were calculated in the mass accuracy range of ±0.5 ppm by considering the following elements: ^1^H (0 to 90), ^12^C (0 to 60), ^13^C (0 to 1), ^16^O (0 to 35), ^14^N (0 to 4), ^32^S (0 to 2), ^34^S (0 to 1). The criteria summarized in Koch *et al*.^46^ were applied for formula assignment. For the final dataset we focused on ions with a relative intensity (normalized to the highest peak in each spectra) of ≥3%, corresponding to a signal to noise ratio of ≥6.

## Additional Information

**How to cite this article:** Lin, Y.-S. *et al*. Near-surface Heating of Young Rift Sediment Causes Mass Production and Discharge of Reactive Dissolved Organic Matter. *Sci. Rep.* **7**, 44864; doi: 10.1038/srep44864 (2017).

**Publisher's note:** Springer Nature remains neutral with regard to jurisdictional claims in published maps and institutional affiliations.

## Supplementary Material

Supplementary Information

## Figures and Tables

**Table 1:** Sample location, site description, and bottom-water DOC concentrations.

Dive number	Core number	Latitude (N)	Longitude (W)	Water depth (m)	Site name and description	Bottom-water^a^ DOC (μmol L^−1^)	Published reference
4562	—	27° 00.461	111° 24.526	2012	Megamat: Large white mat	289 ± 4.6	34
4563	—	27° 00.474	111° 24.425	2009	Patch of white, yellow and orange mat	303 ± 2.5	—
4565	—	27° 00.696	111° 24.265	2010	Cathedral Hill: yellow and white sediment surface near a massive structure of hydrothermal deposits	2112 ± 13.6	34
4567	27	27° 00.542	111° 24.489	2011	Reference site; non-hydrothermal brown sediment	698 ± 6.9	40
4568	1	27° 00.443	111° 24.543	2001	Patch of orange and white Beggiatoaceae mat at the base of a steep structure, backed by *Riftia*	N.A.^b^	—
4569	6, 14, 17	27° 00.470	111° 24.431	2009	Marker 14 Mat: Transsect across orange mat, white mat, and bare sediment	N.A.	13,40
4570	—	27° 00.470	111° 24.428	2010	Large orange and white Beggiatoaceae mat	111 ± 2.4	—

^a^Bottom-water samples were taken at least two meters above the seafloor by Niskin bottles mounted on the HOV *Alvin*.

^b^N.A. = not available.

**Table 2:** Bulk dissolved nitrogen properties and the mole percent composition of the 14 amino acids in the sediment slurries of the heating experiments after 191 days of incubation, with the corresponding DI values calculated from the amino acid data.

Parameter	Time zero	12 °C		50 °C		90 °C	
		Non-sterilized	Sterilized	Non-sterilized	Sterilized	Non-sterilized	Sterilized
*Bulk properties*							
Total dissolved nitrogen (TDN; μmol L^−1^ fluid)	329	427 (27)	385 (10)	3,363 (48)	1,888 (27)	15,788 (123)	18,662 (696)
THDAA (μmol L^−1^ fluid)	5.2	0.7 (0.01)	22 (7)	34 (2)	261 (74)	4,345 (872)	3,526 (1043)
THDAA/TDN (mol% of N)	1.7	0.2 (0.1)	5.9 (3.7)	1.1 (0.5)	14.4 (5.1)	28.1 (11.1)	19.8 (13.1)
*Rel. contribution of individual THDAA (%)*							
THR	8.1	0.0 (0)	2.1 (1.8)	8.2 (0.2)	6.8 (1.1)	0.9 (0.3)	0.2 (0.1)
ARG	1.9	0.0 (0)	1.9 (1.5)	1.7 (0.1)	1.3 (0.1)	0.5 (0.1)	1.1 (0.0)
ASP	14.9	0.0 (0)	2.0 (2.0)	16.2 (0.6)	7.5 (0.7)	7.0 (0.4)	0.4 (0.2)
GLY	25.8	100.0 (0)	34.6 (0.8)	34.8 (0.2)	30.1 (0.5)	28.7 (0.3)	32.9 (0.9)
VAL	2.8	0.0 (0)	4.5 (1.6)	0.0 (0.0)	5.6 (0.9)	6.2 (0.7)	8.5 (0.0)
ALA	20.4	0.0 (0)	22.7 (9.9)	20.4 (0.6)	16.1 (1.4)	17.7 (0.5)	27.1 (1.1)
SER	7.6	0.0 (0)	12.4 (2.7)	11.0 (0.2)	12.0 (1.9)	3.9 (0.2)	0.5 (0.3)
GLU	13.3	0.0 (0)	16.8 (1.2)	6.7 (1.5)	7.0 (1.9)	19.9 (1.2)	14.5 (0.9)
MET	2.2	0.0 (0)	1.6 (1.6)	0.2 (0.1)	4.8 (0.7)	1.7 (0.0)	2.0 (0.1)
PHE	0.0	0.0 (0)	0.0 (0.0)	0.0 (0.0)	1.2 (0.1)	2.1 (0.3)	1.6 (0.0)
ILE	0.9	0.0 (0)	0.4 (0.4)	0.0 (0.0)	3.1 (0.4)	3.5 (0.5)	4.7 (0.0)
HIS	0.1	0.0 (0)	0.0 (0.0)	0.7 (0.7)	0.1 (0.0)	0.3 (0.0)	0.1 (0.1)
LEU	1.9	0.0 (0)	1.1 (1.1)	0.0 (0.0)	4.2 (0.2)	4.6 (0.3)	5.5 (0.1)
TYR	0.0	0.0 (0)	0.0 (0.0)	0.0 (0.0)	0.0 (0.0)	2.9 (0.3)	1.0 (1.0)
**DI**	**−2.2**	**−2.7** (**0.0**)	**−1.8** (**0.8**)	**−3.1** (**0.1**)	**−0.8** (**0.1**)	**0.1** (**0.0**)	**−0.4** (**0.2**)

Numbers in the parentheses are half of the range of duplicate incubations.
