# Supplementary material for: Near-surface Heating of Young Rift Sediment Causes Mass Production and Discharge of Reactive Dissolved Organic Matter
Source: Sci Rep. 2017 Mar 22;7:44864. doi: 10.1038/srep44864 (PMC5361187; doi:10.1038/srep44864)
Supplement: Supplementary Information [file srep44864-s1.pdf]

Supplementary Information

# Near-surface Heating of Young Rift Sediment Causes Mass Production and Discharge of Reactive Dissolved Organic Matter

Yu-Shih Lin, Boris P. Koch, Tomas Feseker, Kai Ziervogel, Tobias Goldhammer,

Frauke Schmidt, Matthias Witt, Matthias Y. Kellermann,

Matthias Zabel, Andreas Teske, Kai-Uwe Hinrichs

**Table S1.** Numbers of identified formulas and intensity weighted average values of molecular weight ($m/z_{wa}$), molar oxygen-to-carbon ratio ($O/C_{wa}$), hydrogen-to-carbon ratio ($H/C_{wa}$), and double bond equivalents ($DBE_{wa}$).

| | Time zero | Non-sterilized incubation, 192 days | | |
|---|---|---|---|---|
| | | 12 °C | 50 °C | 90 °C |
| ***N*(all identified formulas)** | **2215** | **3942** | **4366** | **5224** |
| ***N*(CHO)** | **937** | **1423** | **1238** | **1050** |
| $(m/z)_{wa}$ | 466.8 | 445.0 | 461.6 | 428.1 |
| $(H/C)_{wa}$ | 1.31 | 1.29 | 1.43 | 1.54 |
| $(O/C)_{wa}$ | 0.46 | 0.48 | 0.41 | 0.38 |
| $DBE_{wa}$ | 8.8 | 8.6 | 7.6 | 6.1 |
| ***N*($CHON_{1-2}$)** | **801** | **1616** | **1379** | **1526** |
| $(m/z)_{wa}$ | 461.2 | 442.2 | 463.2 | 421.2 |
| $(H/C)_{wa}$ | 1.24 | 1.23 | 1.32 | 1.23 |
| $(O/C)_{wa}$ | 0.46 | 0.46 | 0.41 | 0.33 |
| $DBE_{wa}$ | 9.9 | 9.6 | 9.3 | 10.2 |
| ***N*($CHON_{3-4}$)** | **270** | **458** | **465** | **1150** |
| $(m/z)_{wa}$ | 489.6 | 490.4 | 496.6 | 443.9 |
| $(H/C)_{wa}$ | 1.13 | 1.13 | 1.19 | 1.42 |
| $(O/C)_{wa}$ | 0.42 | 0.44 | 0.38 | 0.31 |
| $DBE_{wa}$ | 12.4 | 12.3 | 12.1 | 9.2 |
| ***N*(CHOS)** | **193** | **320** | **784** | **450** |
| $(m/z)_{wa}$ | 444.4 | 419.8 | 474.2 | 432.8 |
| $(H/C)_{wa}$ | 1.51 | 1.49 | 1.51 | 1.50 |
| $(O/C)_{wa}$ | 0.44 | 0.50 | 0.43 | 0.37 |
| $DBE_{wa}$ | 6.3 | 5.6 | 6.4 | 6.4 |

**Table S2.** List of parameters, coefficients and constants for Equations (1) to (4).

| Equation Symbol | Explanation | Value | Unit |
|---|---|---|---|
| Equation (1): $\log(\Delta DOC_{max})$=m×$T$+b ($R^2$ = 0.9985) | | | |
| $\Delta DOC_{max}$ | difference between maximum and initial DOC concentrations | measured | μmol $g^{-1}$ dry sediment |
| m | coefficient | 0.0392 | |
| $T$ | temperature | experimentally set | °C |
| b | coefficient | −0.8284 | |
| Equation (2): $\frac{P}{A_0} = 1 - e^{-kt}$ ($R^2$ = 0.9267, 0.9284 and 0.8965 for 12, 50 and 90 °C, respectively) | | | |
| $P$ | concentration of product (heat-mobilized DOC), P = $\Delta DOC_t$ = concentration difference in DOC between a specific incubation time point and experimental start | measured | μmol $g^{-1}$ dry sediment |
| $A_0$ | initial concentration of reactant, $A_0$ = $\Delta DOC_{max}$ | measured | μmol $g^{-1}$ dry sediment |
| $k$ | rate constant | 0.01847 (12 °C)<br>0.02456 (50 °C)<br>0.06209 (90 °C) | $d^{-1}$ |
| $t$ | reaction time | experimentally set | d |
| Equation (3): $k = Ae^{-Ea/(RT)}$ ($R^2$ = 0.8857) | | | |
| $A$ | coefficient | 18.1289 | $d^{-1}$ |
| $E_a$ | activation energy | 0.25 | kJ $mol^{-1}$ |
| $R$ | universal gas constant | 8.314 | J $K^{-1}$ $mol^{-1}$ |
| Equation (4): $T(z) = T_s + (T_{bw} - T_s)e^{(z+dz)v\rho_w c_w/\kappa}$ | | | |
| $z$ | subseafloor depth (positive downward) | measured | m |
| $dz$ | depth offset to compensate for inaccurate estimates of penetration depth | 0.01 (HF1)<br>0.08 (HF5) | m |
| $T_s$ | source temperature | 97 (HF1)<br>204 (HF5) | °C |
| $T_{bw}$ | bottom-water temperature | measured | °C |
| $v$ | Darcy velocity (positive downward) | 13.5 (HF1)<br>70.3 (HF5) | m $d^{-1}$ |
| $\rho_w$ | density of pore water | 1025 | kg $m^{-3}$ |
| $c_w$ | specific heat capacity of pore water | 4184 | J $kg^{-1}$ $K^{-1}$ |
| $\kappa$ | thermal conductivity of the bulk sediment | 0.9 | W $m^{-1}$ $K^{-1}$ |

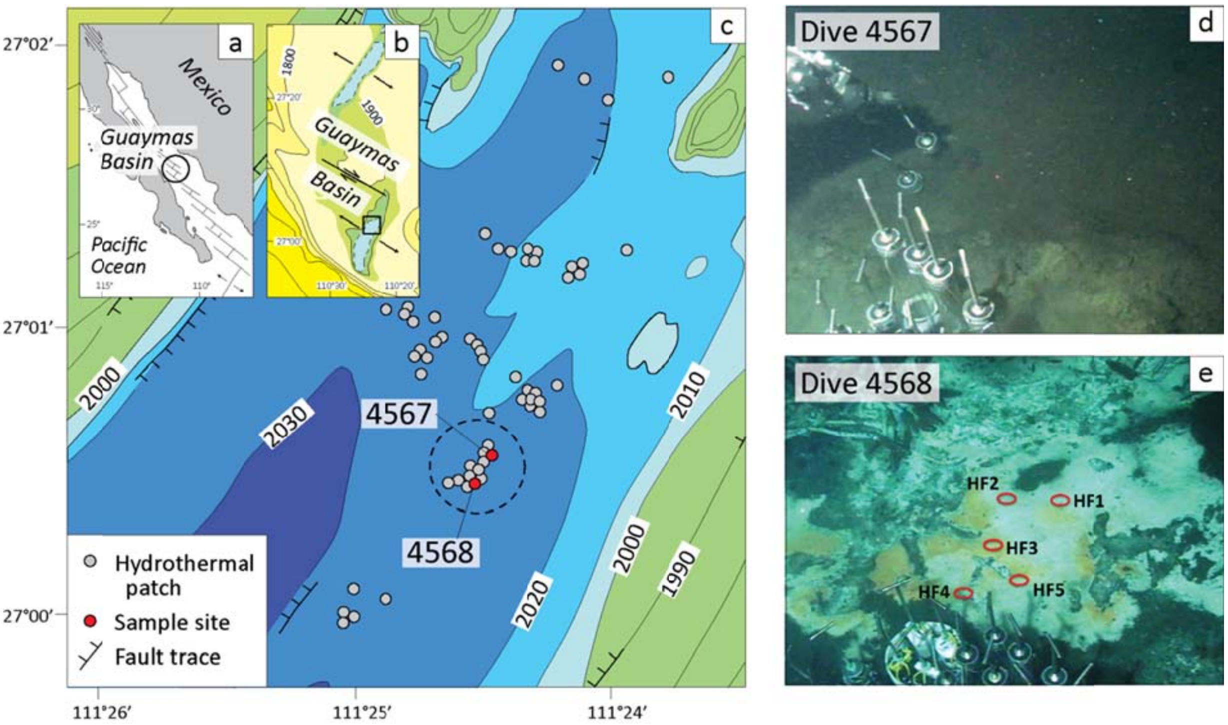


**Fig. S1.** (**a-c**) Study area and sample location (adapted from Lonsdale and Becker[1] and Bazylinski *et al.*[2]). The Guaymas Basin constitutes one of several spreading centers in the Gulf of California that links the East Pacific Rise in the south with the San Andreas Fault leading north. The Guaymas Basin has 3- to 5-km-wide rift valleys, the Northern and Southern Troughs, with floors about 2 km below sea level. Samples were taken from the Southern Trough during the expedition AT15-56 (November-December, 2009) of the RV *Atlantis*. Details of the coring and heat flow measurements have been described in McKay *et al.*[3] Among all of the sediment samples collected by the *Alvin*-operated push corer, we focused on the materials from the reference site and a hydrothermally impacted site, visited during Dives 4567 and 4568, respectively. The distance between these two sites was about 200 m. Base maps reprinted from *Earth and Planetary Science Letters*, 73, P. Lonsdale & K. Becker, Hydrothermal plumes, hot springs, and conductive heat flow in the Southern Trough of Guaymas Basin, 211–225, Copyright (1985), and *Organic Geochemistry*, 12, D. A. Bazylinski, J. W. Farrington & H. W. Jannasch, Hydrocarbons in surface sediments from a Guaymas Basin hydrothermal vent site, 547–558, Copyright (1988), both with permission from Elsevier. (**d-e**) Photos of the sampling sites visited during Dives 4567 and 4568. The spots at which heat flow measurements were carried out were denoted as HF.

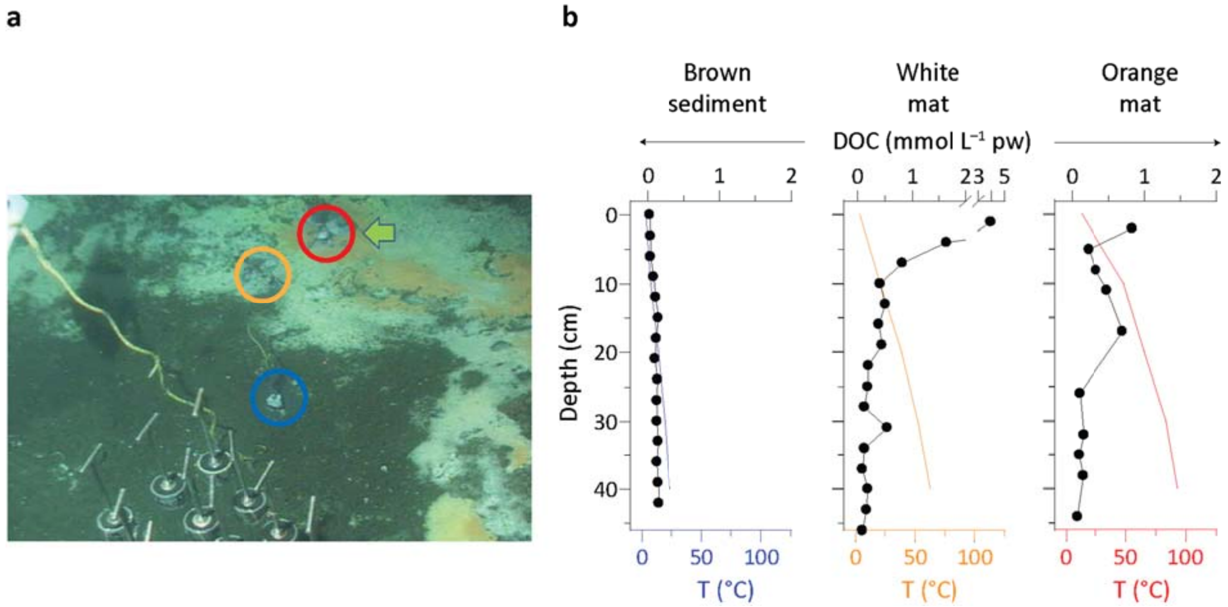


**Fig. S2.** (**a**) Photo of the sampling area during Dive 4569. Push cores were taken from the brown sediment (core 6; blue circle), white mat (core 17; orange circle), and orange mat (core 14; red circle). (**b**) Temperature and DOC profiles of the cores taken from the sites of brown sediment, white mat, and orange mat as marked in (a). Except for the top layers, DOC concentrations in the heated sediment covered by microbial mat were not significantly higher than those of the mat-free sediment. Further geochemical and microbiological characterization of this site is reported in McKay *et al.*[3]

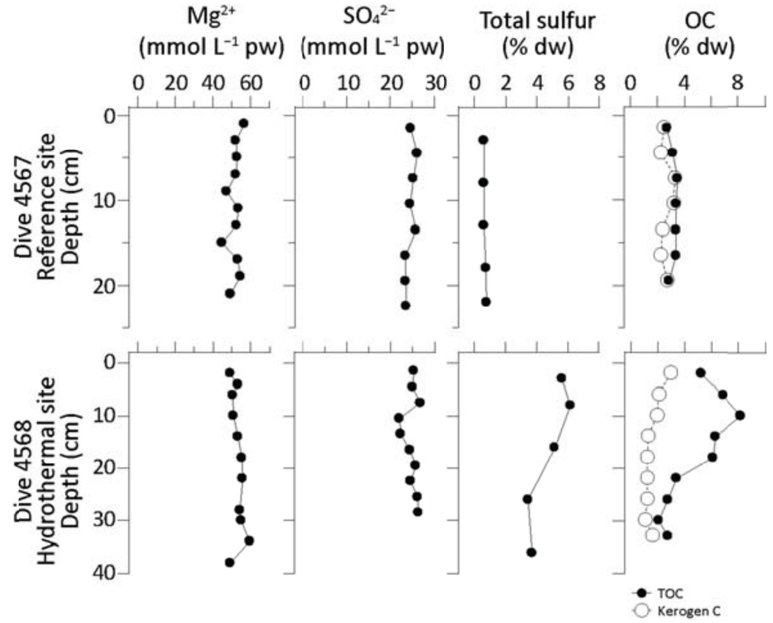


**Fig. S3.** Pore-water magnesium, sulfate, total sulfur content, and organic carbon (OC) content of the reference and hydrothermal sites visited during Dives 4567 (core 27) and 4568 (core 1), respectively. The magnesium and sulfate concentrations, comparable at both sites, are close to seawater concentrations. The hydrothermally impacted sediment was highly enriched in total sulfur compared to the reference site sediment. The total organic carbon (TOC) contents of the heated site were as high as 5–8% dw in the upper 20 cm. Comparison of TOC with the content of kerogen carbon, defined as the non-hydrolyzable and non-extractable fraction of solid-phase carbon, suggests petroleum impregnation at the heated site.

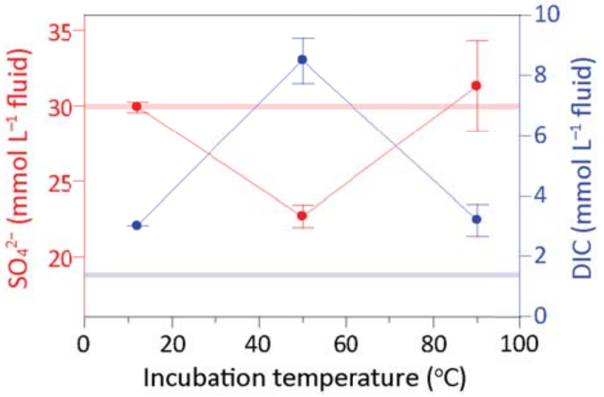


**Fig. S4.** Concentrations of sulfate and dissolved inorganic carbon (DIC) in the experiments of non-sterilized sediment slurries after 191 days of incubation. The increase in DIC was equivalent to remineralization of 1.2% of $TOC_{init}$ (or 0.035% dw). The results are presented as mean and range of duplicate incubations. Red bar, time-zero sulfate concentration; blue bar, time-zero DIC concentration.

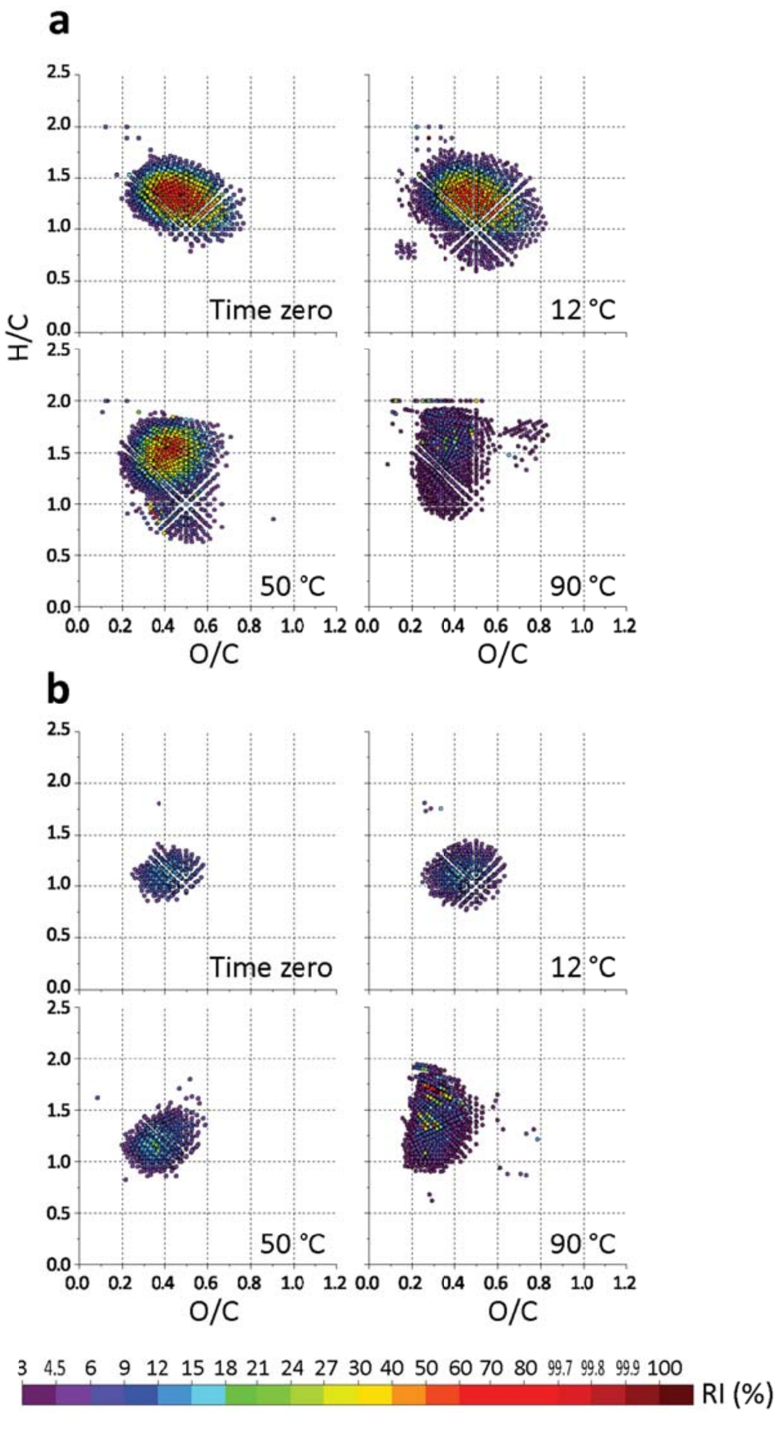


**Fig. S5.** van Krevelen diagrams of (**a**) CHO and (**b**) $CHN_{3\text{-}4}O$ formulas in DOM from the non-sterilized samples of the heating experiment, with the color of the dots indicating relative intensity (RI).

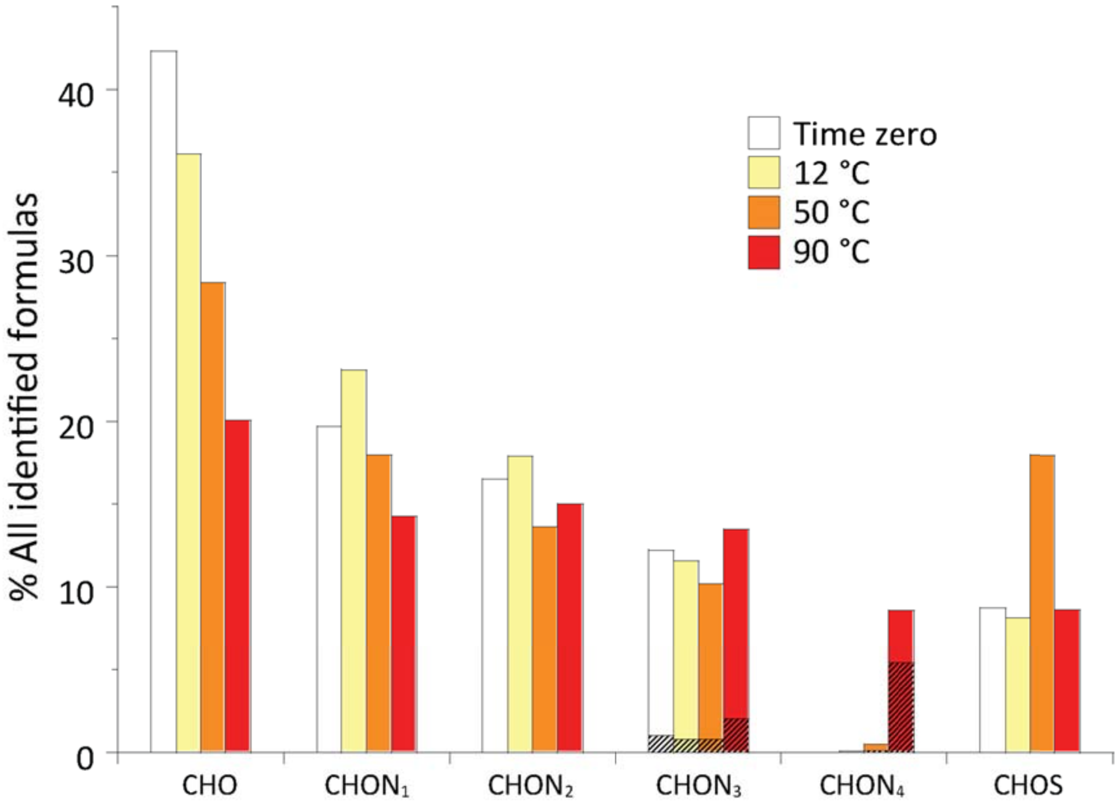


**Fig. S6.** Proportion of major molecular groups in DOM from the heating experiment (non-sterilized sediment slurries). The shaded bars denote the proportion of $CHN_3O$ and $CHN_4O$ species that have matches of tri- or tetrapeptides. The oligopeptide library was computed by matlab.

**References for Supplementary Information**

1. Lonsdale, P. & Becker, K. Hydrothermal plumes, hot springs, and conductive heat flow in the Southern Trough of Guaymas Basin. *Earth Planet. Sci. Lett.* **73,** 211–225 (1985).
2. Bazylinski, D. A., Farrington, J. W. & Jannasch, H. W. Hydrocarbons in surface sediments from a Guaymas Basin hydrothermal vent site. *Org. Geochem.* **12,** 547–558 (1988).
3. McKay, L. J. *et al*. Spatial heterogeneity and underlying geochemistry of phylogenetically diverse orange and white *Beggiatoa* mats in Guaymas Basin hydrothermal sediments. *Deep-Sea Res. I* **67,** 21–31 (2012).
